# Assessment of polyurethane spheres as surrogates for military ballistic head injury

**DOI:** 10.1007/s00414-018-1832-6

**Published:** 2018-03-29

**Authors:** Peter Mahoney, Debra Carr, Nicholas Hunt, Russ J Delaney

**Affiliations:** 1 Centre for Defence Engineering, Cranfield University at the Defence Academy of the United Kingdom, SN6 8LA Shrivenham, UK; 2 Royal Centre for Defence Medicine, ICT Centre Research Park, B15 2SQ Birmingham, UK; 3 Defence and Security Accelerator, Porton Down, SP4 OJQ Salisbury, Wiltshire UK; 4 Forensic Pathology Services, Grove Technology Park, Wantage, OX12 9FA Oxon, UK; 5 South West Forensic Pathology Group Practice, PO Box 388, BS9 0DB Bristol, UK

**Keywords:** 7.62 × 39 mm bullet, Cranial fractures, Ballistic trauma

## Abstract

SYNBONE® spheres were impacted with 7.62 × 39 mm mild steel core ammunition at a mean impact velocity of 654 m/s, SD 7 m/s, to simulate engagement distances of around 50–100 m. The wounds and fracture patterns were assessed by two forensic pathologists familiar with military cranial injury. The overall fracture pattern was assessed as being too comminuted when compared with actual injury. This suggests the SYNBONE® spheres have less utility for simulating military injury than other purposes described in the literature.

## Introduction

The aim of this project was to assess if SYNBONE® spheres (SYNBONE AG, Neugutstrasse 4, 7208 Malans, Switzerland) are suitable for simulating military ballistic head injury at engagement distances of 50 to 100 m.

Much of the ground work in simulating cranial gunshot injury with synthetic models has been done by Thali and colleagues [1–3]. In his initial paper [1], Thali expresses concern that the physical mechanisms behind ballistic trauma are poorly understood. To address this, the group built a synthetic head model using a layered polyurethane sphere (to simulate bone structure), a latex periosteum and a silicone cap to substitute for the scalp. Ten percent gelatine at 4 °C was used as a ‘brain’ fill. The model was shot with a broad range of ammunition (including 7.62 × 51 mm and 7.62 × 39 mm, but mainly 9 × 19 mm full metal jacket, FMJ, Luger) from 10 m and the authors concluded that the injuries created in the model were fully comparable to those seen in real incidents.

Further work with this model included impacting it with 9 mm Luger bullets to explore the underlying mechanisms for entrance wound characteristics [2] and a study of tangential gunshot head injury [3]. In the latter study, the bullets were fired directly at the synthetic skull with the latex periosteum layer (but not the silicone scalp) and found to produce realistic tangential injury and fracture patterns.

More recent work by Taylor and Kranioti has used SYNBONE® spheres to investigate execution style gunshot injuries [4]. The gelatine-filled models were shot at a range of 30 cm with seven different handgun ammunitions with the aim of detecting similarities and differences in wound characteristics for use in future investigations. The authors provide examples of two clinical cases (shot with 0.22LR and 0.45 ACP ammunition) that closely match the corresponding models.

Smith et al. [5] carried out a detailed analysis of the differences between injuries inflicted on a real bone compared to polyurethane bone substitutes. They used both flat plates of synthetic bone (5 mm thick) and spheres (5 and 7 mm wall thickness), and impacted them with a crossbow bolt, a ball fired from a black powder musket, and modern rifle ammunition (0.243″ Winchester Soft Point, velocity 905 m/s, and 7.62 × 51 mm NATO FMJ, velocity 853 m/s). The weapons were fired from a 2 m distance at the targets. They initially compared impacts on flat plates and empty spheres to see if the different shapes affected the response to impact, and compared these with shots into cattle scapulae. There were no gross differences between flat plates and spheres; both showed internal bevelling at the entry site. Differences between the synthetic and real bone are considered later in this paper.

Subsequent work involved spheres filled with 10% gelatine at 4 °C. The secondary and tertiary fracture patterns produced by modern firearms were generally consistent with those seen in published examples of real cranial trauma [5].

## Method

**Fig. 1:**
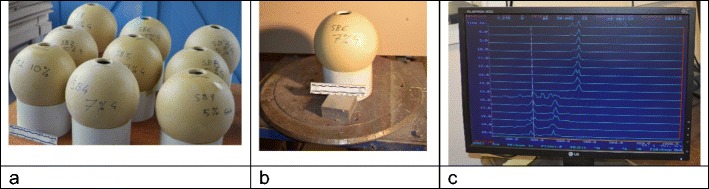
**a** SYNBONE® spheres with gelatine fill; **b** model 6 at the range pre-shooting; **c** screen shot of Doppler radar read out for impact on model 6

Nine SYNBONE® spheres (190 mm diameter, 6 mm wall thickness, thin rubber skin covering outer surface) were filled with ballistic gelatine of either 5,7, or 10% by mass (Fig. 1a). The gelatine was allowed to set at around 17 °C for 24 h. Other work by our group has shown no difference in fracture patterns in a skull model when impacted at a series of temperatures from 4 to 25 °C [6] and no difference with the above gelatine % fills.

The models were taken to the Small Arms Experimental Range, Cranfield University, Defence Academy of the UK (Fig. 1b), and placed 10 m from a no 3 Enfield proof mount fitted with an accurate barrel and shot with 7.62 × 39 mm Ukrainian mild steel core (MSC) ammunition (Soviet State Factory, Lugansk, manufactured 1967; mean impact velocity 654 m/s, SD 7 m/s). The ammunition was downloaded to achieve these velocities simulating engagement distances of around 50–100 m) [7].

Bullet velocity was tracked using a Weibel Doppler (Fig. 1c) and impacts filmed using two Phantom high-speed cameras (V12 from above, sample rate 20,000 frames per second, exposure time 5 μs, resolution 512 × 480; V1212 from the side, sample rate 34,000 frames per second, exposure time 10 μs, resolution 640 × 480).

Models 1–3 were each shot twice to assess their suitability for simulating more than one gunshot injury and assessing if the order of shot impact could be determined as described by Thali [1]. Models 4–9 were each shot once to assess entry and exit fracture patterns from one impact sequence.

The condition of the models in situ post-impact was captured using a Nikon D3200 DSLR camera fitted with an AF-S NIKKOR 18–55 mm lens.

The nine models were then examined by two Home Office forensic pathologists with extensive experience of assessing ballistic injury. The pathologists were invited to score the entry wound, exit wound, and overall fracture pattern using a 4-point Likert-type scale [8] (where 4 = exactly like a real injury, 3 = a lot like a real injury, 2 = a bit like a real injury and 1 = nothing like a real injury) and provide comment as needed. The scores are summarised in Table 1.

**Table 1:** Score of the entry wound, exit wound, and overall fracture pattern using a 4-point Likert-type scale

Model	% gelatine fill	Assessor	Entry wound	Exit wound	Overall fracture pattern
1	10	(a)	2	2	2
		(b)	2	2	2
2	10	(a)	1	2	1
		(b)	1	2	1
3	10	(a)	2	2	2
		(b)	1	2	2
4	7	(a)	1	1	1
		(b)	1	2	1
5	7	(a)	1	1	1
		(b)	1	2	1
6	7	(a)	1	2	1
		(b)	1	2	1
7	5	(a)	1	2	1
		(b)	1	2	1
8	5	(a)	1	2	2
		(b)	1	2	1
9	5	(a)	1	2	1
		(b)	1	2	1

## Results

### High-speed videos (HSV)

**Fig. 2:**
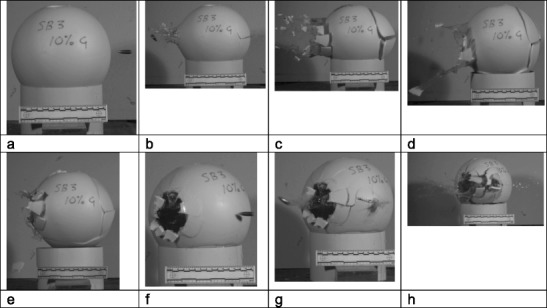
Model 3, 5% gelatine fill, V1212 impact sequence viewed from side **a** pre-impact shot 1; **b** bullet exit, fractures developing entry and exit; **c** further fracture development with temporary cavity expansion; **d**–**e** fragments drawn back in by elasticity of the latex ‘periosteum’; **f** pre-impact shot 2; **g** bullet 2 exit; **h** further fracture development

**Fig. 3:**
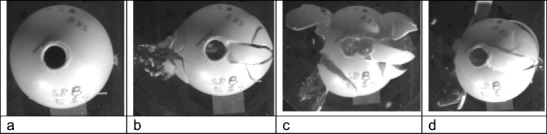
Model 8, 5% gelatine fill, V12 impact sequence viewed from above **a** impact splash visible on right hand side of frame; **b** bullet exit and fracture development, entry and exit sites; **c** disruption of sphere with temporary cavity formation in the gelatine fill; **d** collapse down of temporary cavity with many of the fragments having been retained by the latex ‘periosteum’ dropping back into place

**Fig. 4:**
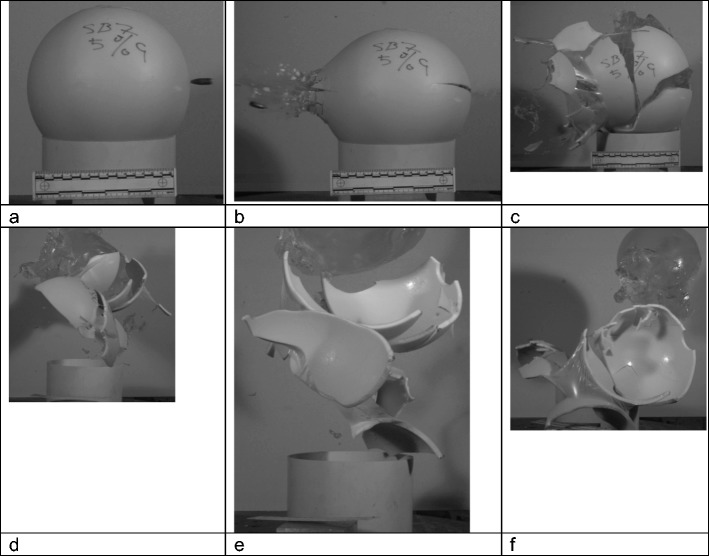
Model 7, 5% gelatine fill, V1212 Impact sequence viewed from side **a** pre-impact; **b** bullet exit; fractures developing at both entry and exit sites; **c** sphere breaks up, latex ‘periosteum’ holds majority of fragments together; **d**–**f** gelatine fill ejected as the sphere breaks up

Example impact sequences taken from the high-speed cameras are shown in Figs. 2, 3, and 4. All HSV triggered and captured the impacts except the V1212 side view for model 4; the overhead view was recorded.

There were two distinct series of events. For models 1, 3, 6, and 8 and the first shot into model 2, the fractures developed as illustrated in Figs. 2 and 3. Fractures spread from both the entry and exit sites but the main fragments were drawn back together by the extendable latex ‘periosteum’. With models 4, 5, 7, 9 and the second shot into model 2, the sphere ruptured and the gelatine fill was expelled (Fig. 4), imitating the ‘Krönlein shot’ [9]. From review of the high-speed videos, the impact sites are not obviously different in the two groups and bullets can be seen to have yawed within the material and exited sideways (Figs. 2b and4b) in an example from each group, although the fractures are more extensive and the integrity of the sphere lost in the models where the contents are completely expelled.

There were also no obvious differences in the fracture patterns seen on the HSV when the spheres with the different gelatine concentrations were compared.

### Pathologist assessment

From Table 1, it can be seen that the most of the entry wounds only scored 1. Although the entry sites displayed bullet wipe and six models had internal beveling, the overall view was that they were too fractured when compared with real incidents and not realistic. The assessors were able to distinguish the different impacts in models 1–3 (model 3 was noted to have a good example of a key hole injury pattern from the second bullet impact) and the order in which they had occurred from the intersecting fracture lines.

The exit wounds scored marginally better but the overall view was that they were too comminuted. External beveling was found in three models (3, 7, and 9), but in others, the exit elements were so fragmented that this could not be assessed.

The overall fracture patterns were judged as being too comminuted when compared with actual military head injury.

The pathologists also noted that differences in bone thickness and the structure within skulls and anatomically correct models does influence fracture patterns as discussed by Fenton et al. [10].

## Discussion

SYNBONE® spheres have been successfully used to simulate ballistic injury by a number of authors. Smith [5] found that the models produced different fracture patterns when impacted by the three projectile types described above, and the black powder carbine did produce a realistic key hole defect from a tangential impact, similar to that described by Thali [3]. Taylor and Kranioti [4] noted differences in the entry wound characteristics between the ammunition types tested with ‘*entrance wound radius ...positively correlated with the caliber dimension*’ and ‘the *number of radiating and concentric fractures is also increasing with the caliber dimension’* [4].

There have been a number of observations regarding how the models differ from real injury. Smith [5] noted that the exit fracture patterns were different from real bone and described *‘stepped fractures where the radius of defect varied widely forming jagged corners around margins, unlike usually rounded/ovoid shapes in real bone’.*

Taylor and Kranioti [4] also noted that the exit wounds in their model were larger than real injury.

The eviscerating injury seen in Fig. 4 was first described by the Swiss Surgeon Rudolf Ulrich Krönlein in relation to close range gunshots with the 1889 Swiss repeating rifle [9]. This effect was also seen by Thali et al. [1].

Our experience using 7.62 × 39 mm ammunition at simulated engagement ranges is that the bony injuries produced in our models were too comminuted and fractured in comparison with contemporary military bony injuries reviewed by the pathologists. This suggests that the model has less utility for this purpose than when used in the tests described by others [1–5]. Of note, two of the 10% gelatine fill spheres had marginally higher scores when compared with the other fills, although the number of replicates for each experiment is small.

## Conclusion

SYNBONE® spheres were assessed for their suitability in simulating military ballistic head injury at engagement distances of 50 to 100 m. Although the overall number of replicates was low (*n* = 9), the impression was that the fractures produced were too comminuted when compared with recent military injury. Further work is ongoing to assess other materials for replicating these injuries.

### Caveats

This experiment only used one ammunition type simulating a particular engagement range. Different results may be obtained with other ammunition and impact velocities.
